# Case Report: Targeted Therapy for Metastatic Solid Pseudopapillary Neoplasm of the Pancreas With *CTNNB1* and *PTEN* Mutations

**DOI:** 10.3389/fonc.2021.729151

**Published:** 2021-10-18

**Authors:** Xinbo Wang, Daojun Zhu, Wei Bao, Min Li, Sizhen Wang, Rongxi Shen

**Affiliations:** ^1^ Research Institute of General Surgery, Jinling Hospital, Nanjing University Medical School, Nanjing, China; ^2^ Department of Clinical Pathology, Jinling Hospital, Nanjing University Medical School, Nanjing, China

**Keywords:** targeted therapy, solid pseudopapillary neoplasm (SPN), metastasis, everolimus, sunitinib, pancreas

## Abstract

**Background:**

Solid pseudopapillary neoplasm (SPN) of the pancreas shows an indolent clinical behavior in cases undergoing surgical resection. The efficacy of combination therapy in the metastatic extrapancreatic SPN treatment remains largely unknown and a clinical challenge.

**Case Presentation:**

We report a case of a metastatic pancreatic SPN in a 45-year-old woman who presented with an aggressive peritoneal dissemination and hepatic metastases and still showed an indolent clinical course with combination therapy with repeated surgery and targeted therapy. Although the follow-up effect remains to be seen, this is the first report of practical experience of the targeted agents sunitinib and everolimus in metastatic SPN tumors based on the mutation status of *PTEN* (c.379G>A; p.G127R) and *CTNNB1* (c.98C>G; p.S33C). To our knowledge, the *PTEN* variant identified in this case has not been previously reported in SPN.

**Conclusion:**

Evidence on variant genetics indicates that future molecular studies may not only help to explain the mechanism of SPN occurrence and development but are also more likely to direct to future precision treatments.

## Introduction

Solid pseudopapillary neoplasm (SPN) of the pancreas is a rare low-grade malignant neoplasm that usually occurs in young women. SPN has an indolent clinical behavior even for cases of large tumor size, and long-term prognosis following surgical resection is generally excellent for both localized and distant disease (1). Patients with SPN occasionally present with liver metastasis or invasion into adjacent organs, relapse immediately after surgery, or cannot undergo surgical resection (2). Combination therapy could help stave off this particularly refractory SPN that arises in response to aggressive surgery. An important support for multidisciplinary comprehensive treatment strategy may be rooted in recent research on cytogenetic analyses and specific molecular pathways of SPN (3, 4).

To the best of our knowledge, the therapeutic value of targeted therapy compared with surgery and adjuvant therapy, as well as their combination, has never been evaluated in patients with metastatic SPN of the pancreas. In this study, we first report a metastatic pancreatic SPN case that presented with refractory peritoneal dissemination and hepatic metastases and maintained stable disease by the targeted agents sunitinib and everolimus based on the mutation status of *PTEN* and *CTNNB1*.

## Case Presentation

**Figure 1:**
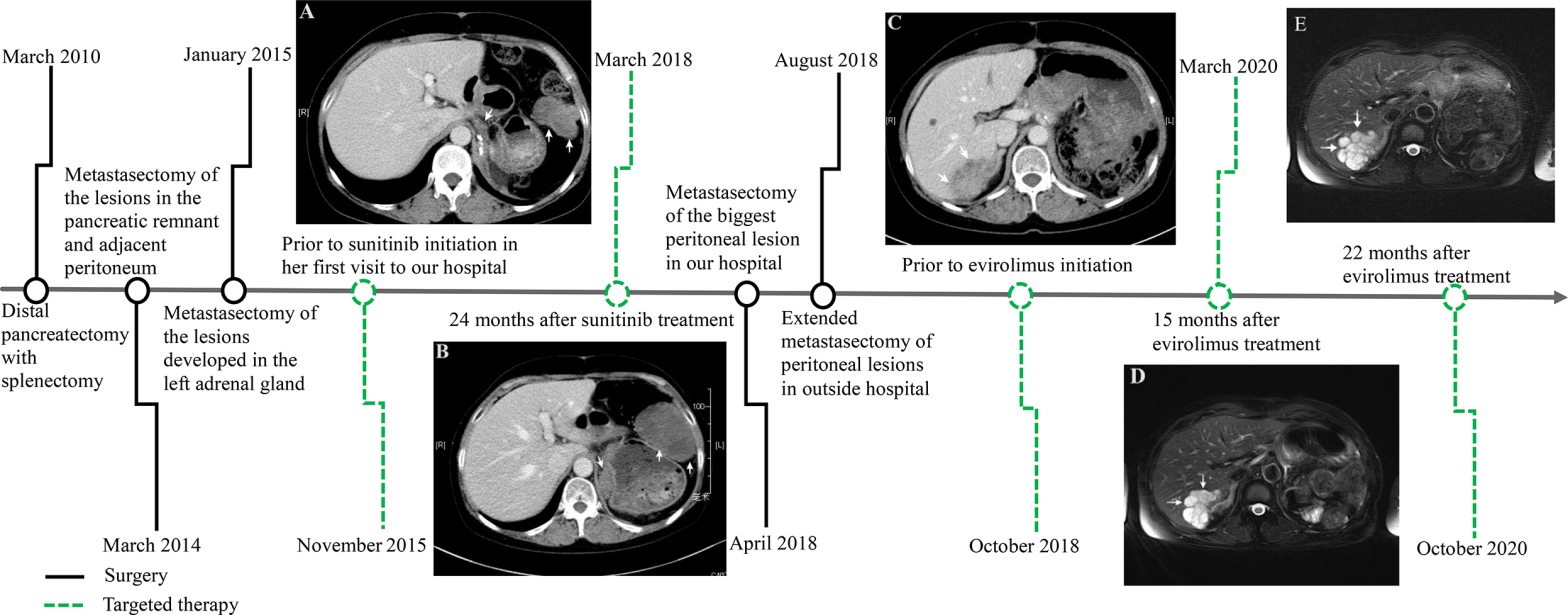
Time axis of repeated surgery and targeted therapy for the metastatic pancreatic solid pseudopapillary neoplasm. Computed tomography scan showing multiple tumors in the peritoneum and retroperitoneum (white arrow) prior to sunitinib treatment after repeated surgery **(A)**. Tumors rapidly increased in size 24 months after sunitinib treatment **(B)**. There was a 52×36 mm, well-defined mass with heterogeneous contrast enhancement in the right lobe of the liver (white arrow) prior to evirolimus treatment after extended metastasectomy **(C)**, which maintained tumor volume stability 15 months **(D)** and 22 months **(E)** after evirolimus treatment.

The patient was a 45-year-old woman who received a distal pancreatectomy with splenectomy for a huge mass, encapsulated solid mass with cystic components (measuring 100 × 70 mm) in the body and tail of the pancreas in the local hospital on March 10, 2010. The histologic examination established the presumptive diagnosis of a well differentiated pancreatic neuroendocrine tumor (pNET). Immunohistochemical analysis showed positive staining for synaptophysin (Syn) and negative for cytokeratin (CK), and chromogranin A (CgA). No adjuvant therapy was administered. Metastasectomy of the lesions (maximum size of 50 × 40 cm) in her pancreatic remnant and adjacent peritoneum was performed with lymph node dissection on March 3, 2014. The revised diagnosis was metastases of low-grade pNET accompanied with SPN immunophenotype. The specimen was positive for Syn, CD56, vimentin, progesterone receptor (PR), and β-catenin immunostaining, whereas negative for CgA. The second metastasectomy was performed to remove recurrent nodules of the left adrenal gland (maximum size of 20 × 15 cm) on January 8, 2015 (**Figure 1**).

During the next 10 months the patient suffered some additional metastatic recurrences (maximum size of 55 × 35 cm) in her left adrenal gland and peritoneum (**Figure 1A**) before her first visit to our hospital on November 17, 2015. The patient participated in a nation-wide clinical study, approved by our hospital’s Ethics Committee, to evaluate the efficacy and safety of sunitinib in the treatment of metastatic pNET in January, 2016. Meanwhile, the pathological features of the tumors from the first three operations were carefully reviewed in the glass slides and paraffin blocks by our own pathologists. All of the tumors were composed of relatively monomorphic polyhedral cells with hyalinized fibrovascular cores. Neoplastic cells were discohesive with round to oval nuclei. Mitotic figures were rare. The growth pattern of the tumor was heterogeneous, with a combination of solid and pseudocystic structures in varying proportions, even in the primary tumor. Sparse vascular invasion was found in the primary tumor with Ki67 index <1%. Immunohistochemical analysis including CD-56, PR, CD10, E-cadherin, and β-catenin was re-performed in the primary and recurrent tumors. All of the tumors were immunopositive for β-catenin (nuclei), CD10, vimentin, PR and Syn and immunonegative for E-cadherin. According to recently published data, a particular dot-like paranuclei expression of CD99 appears to be highly unique for SPN, and this distinctive staining pattern was present in this case. Even in the primary tumor, CD99 expression was punctate and granular positive in the cytoplasm (3+). CD99 accompanied by β-catenin definitely established the diagnosis of SPN in the case (5). The patient was finally diagnosed with a metastatic pancreatic SPN with low-grade malignant potential. She rejected the reoperation for fear of harm from multiple surgeries. According to RECIST 1.1 (response evaluation criteria in solid tumors), the patient’s abdominal metastatic tumors were in a stable state, and given that patients did not have any severe comorbidities while taking sunitinib, the patient continued targeted therapy during the following 2 years.

**Figure 2:**
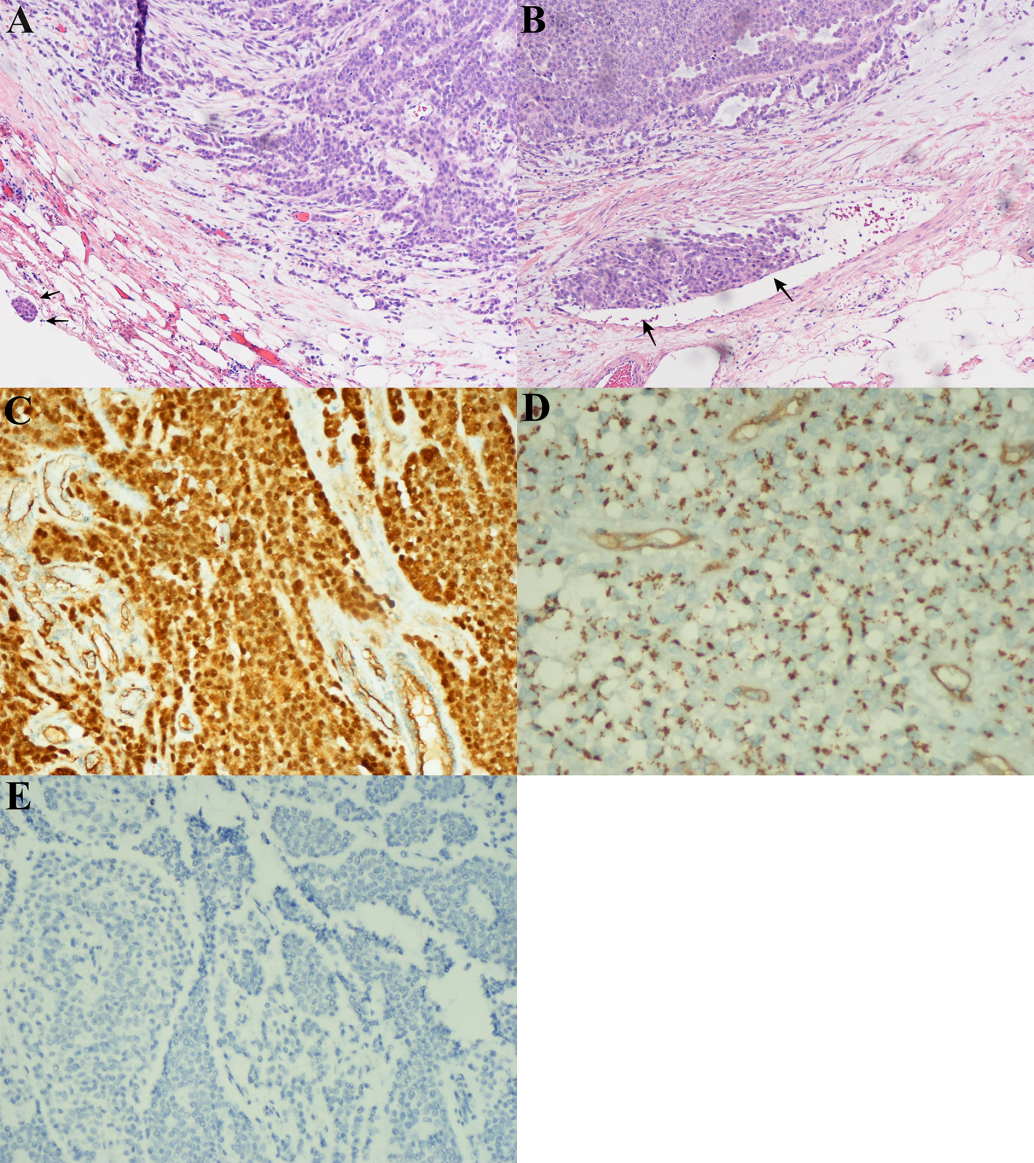
Microscopic and immunohistochemical findings in peritoneal lesions of the metastatic solid-pseudopapillary neoplasms (SPN). **(A)**, The tumor had a heterogeneous growth with predominantly pseudopapillary pattern (hematoxylin-eosin, original magnification ×200). Note the capsular invasion (black arrow). **(B)**, Vascular invasion (black arrow). **(C)**, Nuclear positivity for β–catenin. **(D)**, Dot-like paranuclei positivity for CD99. **(E)**, Negative for chromogranin A (CgA) expression in immuonstaining.

In the first quarter of 2018, the patient presented with some new metastatic lesions in the peritoneum, pelvis, and retroperitoneum, and the earlier lesions increased in size significantly (**Figure 1B**). To obtain the evolutionary features of the tumor immune phenotype, metastasectomy of the biggest peritoneal lesion (maximum size of 75 × 55 cm) was performed on April 18, 2018. The pathological analysis confirmed the metastatic nature of the SPN as capsular with very few capsular and vascular invasions (**Figure 2**). Grossly, the cross-section of the peritoneal metastatic tumor revealed a round, well demarcated mass measuring 75 × 50 mm that consisted of a mixed component containing focal hemorrhage. The tumor showed nested-to-diffuse growth of more poorly cohesive monomorphic cells with scattered nuclear atypia and fibrovascular stalks in which mitosis was sparse with the Ki-67 index 5-10%. The cytologic features also included characteristic myxoid clear material surrounding the papillae, the presence of cercariform cells and sparse mitotic figure. Regional cystic degeneration and haemorrhage were observed. Focal solid areas extended towards the surrounding fat tissue with prominent capsular and vascular invasion. No significant nuclear atypia, abundant necrosis, or high mitotic rate was found. The lesion showed typical immunohistochemical features for SPN, such as nuclear expression of β-catenin, paranuclei dot-like expression of CD-99, positive findings for CD10 (focal region, 3+), vimentin and PR. Interestingly, Syn was positive as always from the primary to the recurrent tumors. On the presumption of disseminated peritoneal metastasis, an extended metastasectomy was performed to remove the left kidney, left adrenal gland, partial diaphragm muscle, small liver nodules, several peritoneal nodules, and pelvic nodules in an outside hospital on August 21, 2018.

Two months postoperatively, the patient presented with a new metastatic lesion in the right hepatic lobe. She was admitted to our hospital again. Computed tomography (CT) scan revealed a well-defined hepatic encapsulated solid mass with cystic components but no calcification (maximum size of 52 × 36 cm, **Figure 1C**). Genomic DNA was isolated from formalin-fixed paraffin-embedded (FFPE) tissue for next-generation sequencing (NGS) by llumina Genome Analyzer. The patient was positive for *CTNNB1* c.98C>G (p.S33C), *ATM* c.5633C>T (p.S1878L), and *PTEN* c.379G>A (p.G127R) point mutations with a germline mutation in *FANCD2* c.888+1G>T. On December 28, 2018, the patient started treatment with everolimus for targeting *PTEN* mutations. Assessment of response was performed by means of computed tomography (CT) scan or magnetic resonance imaging (MRI), every 12 weeks of therapy, according to RECIST criteria 1.1. At present, the patient does not seem to have any serious discomfort except for grade 1 cutaneous vasculitis. She is well, and without any signs of disease progression. The serial follow-up magnetic resonance imaging examination on March 2020 (**Figure 1D**) and October 2020 (**Figure 1E**) showed that the patient’s tumor had partially resolved and remained stable for more than 22 months after the targeted treatment of everolimus.

It was also noted that the tumor immune phenotype might have a certain degree of evolutionary characteristics over time (**Table 1**). In accordance with the immunohistochemical changes, we subdivided the lesions into early (first to third operations) and late (forth to fifth) lesions. In the early metastatic lesions, the tumors had almost no capsular or vascular invasion with weakly positive immunostaining of β-catenin (nuclei, +). The Ki-67 labeling index was also very low (<1%). In the late lesions, malignant histological features, such as peritumoral infiltration into the fat tissue (from the fourth lesion) and the organ, prominent capsular and vascular invasion were observed (**Figures 2A, B**). The specimen was strongly positive for immunostaining of β-catenin (nuclei, +++, **Figure 2C**). Furthermore, the average Ki-67 labeling index increased sharply (3%, 5–10%, and 15% in the third, fourth, and fifth metastatic lesions, respectively).

**Table 1:** Evolutionary characteristics of clinicopathological data of the tumor.

Operation No.	Recurrence interval (mo)	Site	Capsular invasion	vascular invasion	perineural invasion	β-catenin(nuclei)	Syn	paranuclei dot-like expression CD-99	PR	Ki-67 labeling index (%)
1^◼^	0	Pancreas	N	Sparsely	N	+	+	+/-	Sparsely +	<1
2	48	Pancreas Peritoneum	N	Y	N	+	+	NE	NE	<1
3	58	Left adrenal gland	N	N	N	+	+	NE	+	3
4	97	Peritoneum Grater Omentum Retroperitoneum	Y	Y	N	+++	+++	+++	+	5-10
5	101	Peritoneum Grater Omentum Left adrenal gland Retroperitoneum Pelvis Liver	Y	Y	N	++	+	+	+	15

◼ pathological performance amended by our own pathologist.

NE, not evaluable; N, absent; Y, present; Syn, Synaptophysin; PR, progesterone receptor.

+, local expression; ++, moderate expression; +++, diffusively strong expression; +/-, sparse expression.

## Discussion

As there are no specific staging or grading systems for the malignancy of SPN, predicting its metastatic potential is difficult. The evolutionary characteristics of the tumor immune phenotype in this case might explain the aggressive behavior of this tumor. Consistent to our finding, Walter et al. (6) also found that the Ki-67 proliferation index was 2% in the primary tumor of SPN, 10% in ovarian lesions, and 20% in liver metastases. However, investigating the cause of the genetic variation of SPN may have enabled us to describe the metastatic potential molecular pathways involved in this disease (7). Compelling evidences now demonstrate that differences in the molecular pathology, of otherwise indistinguishable cancers, substantially impact the clinical characteristics of the disease (3). The somatic *ATM* gene mutation (c.5633C>T; p.S1878L) in this patient is a variant of unknown significance (VUS) in ClinVar (8) and thus ineligible for treatment. The germline FANCD2 mutation (c.888+1G>T) is also irrelevant, as the mutation that we found is not registered in common data bases such as LOVD3 (9) and thus it might be considered at best as a variant of undetermined significance. In fact, there are currently no US Food and Drug Administration- (FDA)-approved antitumor drugs or related drug-sensitivity studies for the *ATM* gene mutation and germline mutation in *FANCD2* identified in this patient. We focus on the other 2 significant mutations CTNNB1 (p.S33C) and PTEN (q.G127R).

Activating somatic mutations in the β-catenin gene (*CTNNB1* coding) on chromosome 3p occur in almost 100% of SPNs (10), where CTNNB1 mutations disrupt the phosphorylation and degradation of the β-catenin protein. All are missense mutations leading to loss of binding sites for glycogen synthase kinase-3beta (GSK-3b) phosphorylation, thereby interfering with degradation of the β-catenin protein (5). These mutations lead to cytoplasmic and nuclear accumulation of β-catenin in most SPNs. Deregulation of the pathway is closely linked to various aspects of human carcinogenesis such as cell viability, regulation of cell cycle, epithelial-mesenchymal transition, and maintenance of stemness. Consequently, a genetic program is switched on, leading to the uncontrolled growth, prolonged survival, and acquisition of mesenchymal phenotype (11, 12). A possible pathogenetic mechanism involving the interaction between β-catenin and the activation of cell proliferation machinery may also be hypothesized for SPN, although it needs to be better studied and clarified. By viewing the interconnections among each of the genes with variations in SPN specimens, *CTNNB1* was identified as the core portion in the network to regulate tumorigenesis (13). In addition, there is a growing body of evidence suggesting that Wnt/β-catenin signaling plays an essential role in the immune system. In metastatic melanoma, activation of the Wnt/β-catenin signaling pathway correlates with T cell exclusion (14). These reports suggested that activated Wnt signaling mediates cancer immune evasion and resistance to immunotherapies. Nevertheless, these studies could not identify recurrent genetic alterations in metastatic SPNs, which precluded us from identifying common drivers of SPN progression. Recent findings have also demonstrated that β-catenin, lymphoid enhancer-binding factor 1 (LEF1), androgen receptor (AR), and transcription factor E3 (TFE3), which are all expressed in SPNs, interact with each other by diverse pathways, so they are functionally closely interrelated (15). Using whole-exome sequencing and copy number variation analysis it has recently been demonstrated that in metastatic SPNs, in addition to CTNNB1-activating mutations, inactivating mutations of epigenetic regulators (KDM6A, TET1, BAP1) are present in both primary and related metastases, suggesting a role of these genetic alterations in the metastatic dissemination of SPNs. Conversely, most copy number variations were not shared between primary and metastatic lesions from the same patients (4). All of these findings suggest a complex genetic background for SPNs. Future studies based on the molecular alterations of metastatic SPNs will be able to further unveil the pathologic and genetic enigma of metastatic SPN of the pancreas. There are no FDA-approved drugs available for *CTNNB1* mutations, except for a limited number of clinical trials in endometrial cancer. Slomovitz et al. (16) stated that recurrent endometrial cancer patients with *CTNNB1* mutations responded well to everolimus and letrozole. CTNNB1 mutations were also associated with longer progression-free survival (PFS) for advanced endometrial cancer on temsirolimus (17). The signaling relationship between Wnt and mTOR pathways remains an emergent area of study. There is no similar study on SPN disease.

The phosphoinositide 3-kinase (PI3K)-v akt murine thymoma viral oncogene homolog (AKT)-mechanistic target of rapamycin (mTOR) signaling cascade is one of the most important intracellular pathways that is frequently activated in diverse cancers (18). The first identified genetic mechanism of phosphoinositide 3-kinase (PI3K)-v-akt murine thymoma viral oncogene homolog (AKT)-mechanistic target of rapamycin (mTOR) signaling cascade was the loss of *PTEN* function by point mutation or deletion (19). In many types of tumors, the activation of the PI3K- AKT-mTOR pathway has been known as the relation to tumorigenesis, cancer progression and the acquired resistance to various anti-neoplastic agents. Although rare activating somatic mutations in the *PTEN* gene have been described, loss of *PTEN* expression is strongly associated with the presence of invasive carcinoma and poor survival in patients with intraductal papillary mucinous neoplasm of the pancreas (20). *PIK3CA/PTEN* genomic aberrations have been suggested to be strong predictors of everolimus sensitivity (21). The *PTEN* variant identified in this case has not been previously reported in SPN.

Based on the above, considering that our patient has a *CTNNB1* mutation and a non-functional *PTEN* product, it is reasonable to hypothesize that targeting mTOR may provide an effective strategy to regulate SPN tumorigenesis in this patient. This can also explain why she was partially relieved for more than 2 years from refractory metastatic SPNs after five surgeries. What confused us is that this patient actually had maintained stable conditions for nearly 27 months with the target agent sunitinib, a tyrosine kinase inhibitor (TKI) before her forth operation. After that, not only did the original metastases rapidly increase, but new peritoneal metastases appeared. Sunitinib, which has been used to suppress angiogenesis in cancer patients (22), has not been previously reported in patients with SPN. *In vitro* studies showed that inhibition of endogenous PTEN in cultured endothelial cells enhances vascular endothelial growth factor (VEGF) signaling (23). However, clinical reports describe cases in which, after administration of sunitinib, tumor relapse has occurred with severe growth and increased metastatic behavior (24). Treatment with sunitinib led to upregulation of vegfaa in wild-type zebrafish embryos and to a further upregulation of vegfaa expression in mutant embryos lacking *Pten* (25), which may result in hyperproliferation of endothelial cells, hence, explaining the tumor relapse after sunitinib treatment. The signaling relationship between Wnt and mTOR pathways remains an emergent area of study (26); however, this molecular relationship has not been explored in SPN disease. We speculate that combined treatment with a PI3K inhibitor and sunitinib suppresses hypervascularization without enhancing vegfaa expression, suggesting a new approach for therapeutic intervention in VEGFR-signaling-dependent tumors. Combination treatment with TKI and mTOR inhibitors has been evaluated in several phase I trials to date (27). Further investigation using other genomic candidates and new-generation mTOR inhibitors is also warranted in patients with treatment-refractory cancer (28). This study suggests that by targeting the Wnt/β-catenin and the PI3K-AKT-mTOR pathways, it is possible to understand if that would either kill more tumor cells or further delay SPN recurrence.

This case report was based on nonstandardized pathologic report of each institutional pathologic examination during the long time period. Therefore, there might be inconsistency in reporting immunohistochemical characters among different specimens in different hospitals, which can easily confuse the readers. We admit that, in the past, many pathologists and clinicians were unaware of pancreatic SPNs and had no interest at all in these rare pathologic conditions; as such, they just focused on the diagnosis rather than pathologic characterization and oncologic outcomes. However, all the pathological features of the tumors from the formalin-fixed paraffin-embedded (FFPE) tissue were carefully reviewed by our own pathologists. Furthermore, the focus of this case report is to suggest that evidence on variant genetics indicates that future molecular studies may not only help to explain the mechanism of SPN occurrence and development but are also more likely to direct to future precision treatments.

## Conclusion

In conclusion, this case depicted the aggressive peritoneal dissemination and hepatic metastases of a metastatic pancreatic SPN that still showed an indolent clinical course when combination therapy with repeated surgery and targeted therapy were applied. Although the follow-up effect remains to be seen, this is the first report of practical experience of the targeted agents sunitinib and everolimus in metastatic SPN tumors based on the mutation status of *PTEN* and *CTNNB1*. Future molecular studies are required to provide more precision tools to guide preclinical and clinical therapeutic development and treatment in metastatic SPN.

## Data Availability Statement

The original contributions presented in the study are included in the article/supplementary material. Further inquiries can be directed to the corresponding author.

## Ethics Statement

The studies involving human participants were reviewed and approved by Jinling Hospital, School of Medicine, Nanjing University. The patients/participants provided their written informed consent to participate in this study. Written informed consent was obtained from the individual(s) for the publication of any potentially identifiable images or data included in this article.

## Author Contributions

XW was involved in the identification, selection, and management of patient cases and wrote and revised the manuscript. DZ, ML, SW, and RS was involved in the management of patient cases. WB performed the histological images analysis and reviewed the manuscript. All authors contributed to the article and approved the submitted version.

## Conflict of Interest

The authors declare that the research was conducted in the absence of any commercial or financial relationships that could be construed as a potential conflict of interest.

## Publisher’s Note

All claims expressed in this article are solely those of the authors and do not necessarily represent those of their affiliated organizations, or those of the publisher, the editors and the reviewers. Any product that may be evaluated in this article, or claim that may be made by its manufacturer, is not guaranteed or endorsed by the publisher.
